# Guidelines for the use and interpretation of diagnostic methods in adult food allergy

**DOI:** 10.1186/s12948-015-0033-9

**Published:** 2015-10-05

**Authors:** Donatella Macchia, Giovanni Melioli, Valerio Pravettoni, Eleonora Nucera, Marta Piantanida, Marco Caminati, Corrado Campochiaro, Mona-Rita Yacoub, Domenico Schiavino, Roberto Paganelli, Mario Di Gioacchino, On behalf of the Food Allergy Study Group (ATI) of the Italian Society of Allergy, Asthma and Clinical Immunology (SIAAIC)

**Affiliations:** SS Allergology and Clinical Immunology, S. Giovanni di Dio Hospital, Florence, Italy; Respiratory Diseases and Allergy, University of Genoa, IRCCS AOU S. Martino-IST, Genoa, Italy; Allergology and Immunology Unit, IRCCS Ca’ Granda, Osp. Maggiore Policlinico, Milan, Italy; Servizio di Allergologia, Policlinico Gemelli, Rome, Italy; Allergy Unit, Verona University and General Hospital, Verona, Italy; Department of Allergy and Clinical Immunology, IRCCS San Raffaele Hospital, Milan, Italy; Department of Medicine and Science of Ageing, G. d’Annunzio University, Chieti, Italy; Unit of Allergy and Immunotoxicology, CeSi, “G. d’Annunzio” University Foundation, Chieti, Italy

**Keywords:** Food allergy diagnosis, Skin prick test, Molecular allergens, Molecular-based diagnosis, Challenge test, Basophil activation test

## Abstract

Food allergy has an increasing prevalence in the general population and in Italy concerns 8 % of people with allergies. The spectrum of its clinical manifestations ranges from mild symptoms up to potentially fatal anaphylactic shock. A number of patients can be diagnosed easily by the use of first- and second-level procedures (history, skin tests and allergen specific IgE). Patients with complex presentation, such as multiple sensitizations and pollen-food syndromes, frequently require a third-level approach including molecular diagnostics, which enables the design of a component-resolved sensitization profile for each patient. The use of such techniques involves specialists’ and experts’ skills on the issue to appropriately meet the diagnostic and therapeutic needs of patients. Particularly, educational programs for allergists on the use and interpretation of molecular diagnostics are needed.

## Background

Food Allergy (FA) is an increasingly recognized problem in relation to its prevalence in the general population. In Italy, it corresponds to 8 % of all patients with allergies [1–3] and the broad spectrum of its clinical manifestations, ranging from mild symptoms up to potentially fatal anaphylactic shock (Table 1). FA significantly affects the quality of life of patients and their families [4]. In adults, FA may persist from childhood or may develop at an older age. In the latter case, once established, FA is maintained throughout life, while paediatric FA frequently disappears during adolescence. FA may be responsible for signs and symptoms that occur shortly after consumption of the culprit food (from a few minutes to a few hours). The earlier they arise, the more serious they are. At times, symptoms appear after physical exercise (food dependent exercise induced anaphylaxis, FDEIA) and the ingestion (about 3 h before) of a specific food, which is safely eaten in the absence of exercise [5].

**Table 1:** Main food allergy symptoms

Organs and systems	Clinical manifestations
Respiratory	Oculorhinitis
	Bronchial asthma
	Oedema of the glottis
Skin and subcutaneous tissue	Erythematous rash
	Itching without rash
	Urticaria-angioedema
	Atopic dermatitis
	Eczema
Gastro-enteric	Oral Allergy Syndrome
	Abdominal pains
	Vomiting
	Diarrhoea
Cardiovascular system	Hypotension
	Cardiac arrest
	Anaphylactic shock

FA most commonly affects the skin (atopic dermatitis, urticaria, angioedema, eczema and various skin rashes) [6, 7]. Frequently, gastrointestinal manifestations are associated with cutaneous symptoms. The gut is rarely the only organ affected by food allergy. Symptoms range from dyspepsia and meteorism to colic, diarrhoea (rarely constipation), vomiting, gastroesophageal reflux, up to the most complex malabsorption syndromes, generally due to the infiltration of inflammatory cells in the gastrointestinal mucosa [8–10]. In some cases, mainly in pollen-allergic patients sensitive to molecules homologous to those contained in specific foods, symptoms appear in the form of itching and burning of the oral mucosa, papules or vesicles in the mouth, swelling of the lips and difficulty in swallowing, being defined as oral allergy syndrome [11]. Rhinitis, conjunctivitis, asthma and laryngeal edema are all possible FA manifestations independent from sensitization to inhalant allergens [12].

Each year 4–5/100,000 patients experience an anaphylactic shock, with a cumulative risk equal to 0.5–2 % [13]. Foods are the main cause of anaphylactic shock for children and young adults, whereas for older people, insect stings are mainly responsible. This syndrome is due to the involvement of the cardiovascular system with a drop in blood pressure due to vasodilation and leakage of fluids from the circulation, with systemic consequences [14]. The term anaphylaxis (without shock) is referred to a reaction involving multiple organs, usually the skin, gastrointestinal tract and respiratory system.

There is no consensus on allergy due to food contaminant and additives. Clinicians sometimes report the disappearance of the characteristic symptoms of food allergy after an additive-free diet, despite the fact that there is no scientific evidence on their actual role in causing symptoms [15–17]. In any case, reactions are not mediated by an immunological mechanism and are classified as non-allergic hypersensitivity reactions. There is a possibility that food reactions also stem from some non-protein food component or from other mechanisms, for example, cell-mediated mechanisms. These include reactions to orally ingested nickel, the so-called Systemic Nickel Allergy Syndrome, which is characterized by the appearance of gastrointestinal symptoms (typically meteorism, colic and diarrhoea) and skin manifestations (eczema, urticaria and angioedema) in sites without nickel contact in patients with nickel contact dermatitis, and responds positively to a low-nickel diet [18].

Diagnostic efforts are directed to the identification of the food(s) involved in triggering and/or maintaining the symptoms. This can be achieved by using all available diagnostic methods applied in an appropriate sequence, avoiding non-standardized ones.

The purpose of this document is to define guidelines for the use and interpretation of scientifically validated and recognized diagnostic methods for food allergy.

## Basic concepts of FA

Primary forms of FA are due to a sensitization process caused by ingestion. In the secondary forms, the patient is sensitized by inhalation to allergens containing molecules homologous to those contained in certain foods, whose ingestion may cause symptoms usually in the oral cavity, in the frame of an oral allergy syndrome [19]. Several molecules with different characteristics act as food allergens. Some of them are stable, enduring heating, cooking, storage and digestion (linear epitopes), while others are less stable (conformational epitopes) losing their allergenicity in cooking and preservation [20]. The patient with FA can be sensitized to both labile and stable components. The stability/lability to physical agents (heat, gastric pH, enzymes like protease, pepsin and so on) is a requisite for an allergen to interact with the IgE antibody. Thus, a component sensitive to heat will be virtually absent in a cooked food, while a determinant resistant to heat, pH and peptidase (for example, Lipid Transfer proteins–LTP) will reach the bowel practically intact despite cooking and passage through the gastric and pancreatic digestion [21]. A particular situation arises with the use of antacid drugs that do not allow (or partially allow) the denaturation of acid sensitive proteins, thus resulting in unpredictable symptoms [22]. Other substances can act as “co-factors”, increasing the likelihood of anaphylaxis from food allergens. They include alcohol, non-steroidal anti-inflammatory drugs (NSAIDs), hormonal influences, bacterial or viral or parasitic infections [23] and chemicals [24–26].

The large variety of clinical manifestations (Table 1) and the complexity of allergens often make the diagnosis of FA difficult. A component-specific profile, other than extract-specific, should be used for an optimal definition of the sensitization. In addition, in the specific field of food allergy, it is crucial to discriminate between cross-reactions and co-sensitization, particularly for members of the plant kingdom (“pollen food allergy”), and to more accurately estimate the risk of severe reactions. The sensitization to cross-reactive molecules is relatively rare in childhood but tends to appear during adolescence and remains stable in adults. The recent adoption of individual allergenic molecules (Molecular-Based Diagnosis, MBD) in diagnosis allows for the definition of a more precise IgE profile for the patient, e.g. adding prognostic information related to the possible risk profile of the reaction. Understanding the fine relationships between the results of in vivo and in vitro tests and the patient’s clinical picture is the key for any further clinical decisions.

## Diagnostic methods for food allergy

A correct diagnosis of FA requires an established diagnostic procedure. The first step is always the patient’s history aimed at identifying the suspected relevant food(s) and the relationship between the ingestion of a specific food and the occurrence of symptoms. Then, the dependence of clinical manifestations from an immune mediated reaction must be assessed. This can be done by both in vivo and in vitro tests.

The standardized diagnostic methods are classified into first, second and third level.

### First-level methods

#### Medical history

Medical history is essential in every field of medicine because it allows one to obtain all the information and data that can help to move towards the diagnosis of a certain disease. It comprises the physiological, family (investigating all possible genetic risk factors or any family predisposition) and the past and current medical history. The latter investigates the disorder for which the patient consulted a doctor. In suspected FA, repeated clinical manifestations related to the ingestion of given meals are highly indicative. Medical history should be addressed to clarify (a) the presence or absence of similar symptoms in other people when they consumed the same food(s); (b) the ingested food(s) in the 2–4 h before the onset of symptoms; (c) the allergens that may contaminate food preparation (for example, casein, latex, ovomucoid); (d) the cooking and storage of food; (e) the presence of triggering factors; (f) the existence of other allergies (such as respiratory or skin allergy) or other diseases [27]. A correct diagnostic approach also requires a complete physical examination. When too much time has elapsed from the appearance of symptoms, it could be difficult to identify the offending food, in particular, if the allergen is not easily identifiable or “hidden”. Medical history can be re-evaluated starting from the results of in vitro and in vivo tests, which could demonstrate sensitizations to foods that were initially not considered [28, 29].

#### Skin tests

*Skin prick test (SPT)* The SPT is a well-standardized, simple, cheap and low-risk diagnostic test. It should be the first step performed and both inhalant and food allergens should be tested. Table 2 shows a panel of food allergens to be tested, supplemented, where appropriate, by foods chosen according to patient’s history and dietary diaries, and Table 3 shows the technical procedure to be used. The SPTs to foods have a low specificity with a low positive predictive value. Thus, a positive result, unless confirmed by the clinical data, does not allow for a definitive diagnosis of FA [28–30]. In children, cut-off values for the SPT reaction diameter for certain food allergen (milk: 8 mm, egg: 7 mm, peanut: 8 mm) have been identified but are not universally acknowledged. However, oral food challenges were always positive (100 % specificity) in children with cutaneous reactions of this diameter or above [31, 32]. In general, SPT have an excellent sensitivity with high negative predictive value (>90 %), thus a negative result generally rules out the possibility of an IgE-mediated sensitization. However, this is true only for foods containing stable proteins, such as casein from cow’s milk, egg ovomucoid, albumin and peanut vicilins, which are well represented in the extract. The SPT performed with allergenic extracts containing thermo-labile molecules, such as pathogenesis-related-10 (PR-10) proteins have a low negative predictive value. For these allergens, the prick + prick (P + P) procedures with fresh foods can be useful.

**Table 2:** Food panel for Prick test

Egg	Peach
Peanut	Apple
Beta-lactoglobulin	Cod
Banana	Hazelnut
Carrot	Walnut
Casein	Fish
Bean	Pea
Wheat flour	Chicken
Shrimp	Tomato
Lactalbumin	Rice
Pork	Celery
Corn	Soybeans
Almond	Egg yolk

**Table 3:** Technical procedure for SPT

Apply one drop for each allergen extract to be tested, maintaining a minimum distance of 3 cm between drops on the volar part of the patient’s forearm (5 cm from wrist and 3 cm from the antecubital fossa)
Apply pressure, through a sterile, disposable lancet, to each single allergen, pricking to a depth of 1 mm for each drop, perpendicular to the skin’s surface
Hold for about 3 s with moderate pressure without moving the hand or turning to avoid bleeding
Carefully remove the allergen solution with blotting paper
The same procedure is to be followed to test histamine (10 mg/ml) as a positive control and physiological glycerine as a negative control
Reading of the results: after 15 min from the performance of the test
Interpretation of the test: a positive result is defined by the appearance of a wheal of at least 3 mm in average diameter. Responses to histamine and the negative control should be carefully considered. The latter verifies that the patient does not suffer from dermographism and the former demonstrates a “normal response” to histamine (with no negative interference from drugs or other conditions, such as hypo-reactivity of the skin)

The major limitations of allergen extracts for SPT are represented by (a) the content, because each extract is a heterogeneous mixture composed of major and minor allergenic proteins, and other biologically inactive components such as non-allergenic proteins, glycoproteins and carbohydrates, (b) the production process, because some allergens may undergo partial degradation during the extraction, (c) the cross-reactions, as different biological sources may contain cross-reactive allergens.

An in vivo MBD approach (available in vitro for many molecules, shown in Table 4) is also possible with extracts containing high concentrations of LTP (a gastro- and thermo-stable protein from *Rosaceae*) and palm profilins (Pho d 2, an ubiquitous gastro- and heat labile plant protein). Their use, to complete the diagnostics performed with extracts from whole sources, allow for a more precise assessment of the ingestion risk of the suspected food [33].

**Table 4:** Native or recombinant molecules available for SPT

Molecule	Source
Lactalbumin	Cow’s milk
Beta-lactoglobulin	Cow’s milk
Casein	Cow’s milk
Ovalbumin	Egg white
Ovomucoid	Egg white
LTP (Pru p 3)	Peach
Profilin (Pho d 2)	Palm tree
PR10 (Mal d 1)	Apple (not available in Italy)

*Prick* *+* *prick (P* *+* *P)* P + P is performed with fresh food, in particular vegetables, when the commercial extract is negative (or unavailable) but the clinical history is suggestive. When the food is solid, the technique involves firstly puncturing the fresh food (some allergens are located just under the skin of the fruit) and then the patient’s skin with a lancet according to the SPT standard procedure [34]. When the food is liquid, the technique is the same as in SPT.

P + P has a good diagnostic reliability [35] with high predictive negative values. In the case of a positive result, it must be always taken into account that some foods are rich in histamine and lectins and can produce false positives. Obviously, the use of skin P + P with fresh food is not entirely risk-free and highly sensitive subjects may suffer systemic adverse reactions [36].

#### Atopy patch test (APT)

The APT is performed through the same technique used for common patch testing to identify the responsible hapten in contact dermatitis, and is aimed at assessing the delayed cell-mediated hypersensitivity to foods that may especially occur in children with atopic dermatitis or gastrointestinal reactions to foods. In 2010, the APT was considered an emerging test, like BAT and MBD [37], but subsequent studies did not confirm its diagnostic role to be as important as the other two techniques.

### Second-level methods

#### In vitro assays for total serum IgE (tIgE) and specific IgE (sIgE) to foods

Like SPT and P + P, in vitro tests only certify a sensitization and the interpretation of results is the allergist’s task. Thus, measuring tIgE may be useful in grading allergy conditions, but only when used in combination with other tests. Indeed, tIgE alone has no predictive value in relation to the diagnosis of FA. The assay of sIgE for food extracts is a second-level test in the view of costs. Thus, it should be requested only after skin tests. However, it may still be exceptionally considered a first-level test in those conditions in which SPT cannot be performed (e.g. very young paediatric patients, concomitant antihistamine therapy or skin alterations, risk of systemic reactions). Importantly, an in vivo test is able to detect the biological effects (revealed by wheal, redness, itching, etc.) caused by the presence of sIgE bound to skin mast cells, while the serum test only detects the presence of circulating IgE specific to a particular allergen. It is therefore possible that the results of the two tests are different [38].

Nowadays, quantitative methods with extracts have levels of sensitivity (and negative predictive values) comparable to APT, with high specificity and positive predictive value [37]. The test is suitable to detect the IgE specific for a given allergen, in a quantitative way, in a range between 0.10 and 100 kU/L. As for the SPT reaction diameter, specific IgE levels exceeding a certain value (“diagnostic cut-off”) showed a predictive value of 95 % for a symptomatic allergy [32, 39] (Table 5). Thus, in the presence of a compatible clinical history, sIgE can confirm the diagnosis of FA without requiring further challenge tests. However, the predictive values vary from one study to another. The results are influenced by many variables such as the patient’s age, duration of food allergen avoidance at the time of testing, selection of patients and clinical disorders, and have been validated on non-European test subjects. It is also important to stress that the values of specific IgE <0.10 kU/L does not exclude the possibility of an IgE-mediated allergic reaction and that the confirmation of a negative test, in the case of strong clinical suspicion, can only be achieved with negative SPTs and negative challenge tests.

**Table 5:** Sensitivity, specificity, positive (PPV) and negative (NPV) predictive value of tests for the detection of specific IgE in vitro for the most common food allergens

Allergen	Sensitivity (%)	Specificity (%)	PPV (%)	VPN (%)	Diagnostic cut-off (kUA/l)
Egg	61	95	98	38	6
Milk	57	94	95	53	15
Peanut	57	100	100	36	14
Codfish	63	91	56	93	3
Soybean	44	94	73	82	30
Wheat	61	92	74	87	26

#### In vitro MBD

Diagnostics based on allergenic extracts allow for the identification of the allergen source (e.g. fish, egg, milk, etc.) but not the molecular component to which a patient is sensitized, which can be studied instead through in vitro MBD and therefore used to improve the result of a sIgE test [20]. In vitro MBD uses molecular allergens isolated from a given allergen source (purified or native allergens) or produced by recombinant DNA technology (recombinant allergens) (Tables 6, 7). This approach improves the description of the IgE repertoire against food allergens or their molecular components and explains cross-reactions and their role in FA.

**Table 6:** Major food allergens and components available for molecular diagnostics using ImmunoCAP (or ImmunoCAP ISAC)

Allergens (or allergen source)	Protein family
Cupin superfamily	
Vicilins	Ara h 1 (peanut)
Legumins	Ara h 3 (peanut), Cor 9 (hazelnut)
Prolamin superfamily	
2S albumin	Ber e 1 (brazil nut), Ara h 2 (peanut), Gly m 6
Lipid transfer protein (LTP)	Pru p 3 (peach), Cor 8 (hazelnut), Art v 3 (*Composite*) Jug r 3 (walnut)
Cereal prolamines	Tri 19 (wheat) Tri a 14
Pathogenesis-related (PR) proteins	
PR10: intracellular proteins	Pru p 1 (peach), Api g 1 (celery), Gly m 4 (soy)
PR3: chitinase Class 1	Hev b 11, Hev b 2.6 (latex, banana, avocado)
Profilins	Pru p 4 (peach) (Bet v 2, Phl p 12, Hev b 8)
Cross-reactive carbohydrate determinants	MUXF3 (celery, tomato)
Tropomyosins	Pen a 1 (shrimp)
Calcium binding proteins	
Parvalbumin	Gad c 1 (codfish)
Milk proteins	Bos d 4 (α-albumin), Bos d 5 (β-lactoglobulin), Bos d 8 (casein), Bos d lactoferrin (lactoferrin)
Egg protein	Gal d 1 (ovomucoide)
	Gal d 2 (ovalbumin)
	Gal d 3 (conalbumin)
	Gal d 4 (lysozyme)

**Table 7:** Families of protein carbohydrate molecules mainly involved in food allergy

Molecules associated with allergy to food source (or source allergen)
PR-10 proteins (homologous to Bet v 1)
Non-specific lipid transfer proteins (nsLTP)
Profilins
Storage proteins
Thaumatin-like-proteins (TLP)
Cross-reactive carbohydrate determinants (CCD)
Molecules associated with allergy to food of animal origin
Tropomyosins
Parvalbumins
Caseins
Lipocalin, Family of lysozyme, Family Transferrins, Ovomucoids

The MBD approach should be used to distinguish patients with genuine sensitization towards a food (with high risk of accidental ingestion) from those with co-sensitization, i.e. sensitization to ubiquitous proteins present in pollen (which act as primary sensitizing) and also common in food (with a much lower risk of adverse reaction). Again, it is possible to identify patients characterized by sensitization to food independently by a sensitization to aeroallergens (primary sensitization) and patients with a “pollen-food syndrome”, where the first sensitization occurs via inhalation and the great homology between the allergen of the “first sensitizer” and some food allergens is responsible for the patient’s symptoms presenting as an oral allergy syndrome [40, 41].

Identifying cross-reactions is a further benefit of MBD. The allergist is able to understand whether a single, a few closely related or several widely different food allergen sources should be considered in a dietary approach. The allergist will also be able to assess the risk of a given FA identifying, by in vitro MBD, patients sensitized to “relatively harmless” or potentially very dangerous components [20] that need the prescription of life-saving drugs such as auto-injectable adrenaline together with a strict allergen avoidance. The use of MBD requires allergists to acquire new skills.

Primarily, they need to learn the new allergen nomenclature [42, 43]. International classification ranks the allergenic source first by its scientific name, from which it takes the first three letters of the generic name and the first letter of the species (or two letters when confusion is possible): e.g. apple is scientifically called “*Malus domestica*”: therefore Mal d indicates the allergen source. Adding a number (1, 2, 3 etc.) indicating the chronological order of the identification allows for the classification any allergenic molecules: e.g. for apple the identified molecules are named Mal d 1, Mal d2, Mal d 3, and Mal d 4.

It is also important to know the molecular allergenic content of foods. Some molecules are specific for a given food, allowing the identification of the primary sensitizer, others share common epitopes (antibody binding sites) and the same IgE can induce an immune response to allergenic molecules with similar structures from different allergen sources [33]. In the example of apple, Mal d 3 is an LTP molecule homologous to the LTP of peach, nuts, apricot, cherry, etc. and an exclusion diet should prohibit all these foods, but only according to the patient’s history [44]. Indeed, Mal d 1 is highly homologous to the birch pollen allergen Bet v 1 and characteristically induces an oral allergic reaction [45].

The molecular structure and physiochemical properties of allergens are major determinants of their clinical relevance. For example, LTPs are particularly resistant to high temperature and enzymatic degradation, so cooking and digestive processes are unable to deactivate their allergenic capacity. For this reason, LTP exposure through the gastrointestinal tract may induce sensitization in predisposed individuals and may trigger severe reactions in sensitized patients [46]. The specific patient’s sensitization profile is relevant in terms of risk assessment. In fact, the simultaneous sensitization to peach LTP Pru p 3, Pru p 1 and Pru p 4 in the same patient seems to exert a protective role in comparison with Pru p 3 sensitization alone, as it is associated with less severe symptoms [47]. Similarly, it has been recently shown that in peach-allergic patients with tomato hypersensitivity, sensitization to rPru p3 seems to be a surrogate biochemical marker for a severe tomato allergy, whereas the presence of anti-rPru p 1 IgE may be an indicator of a mild tomato allergy [48].

Profilins are pan-allergens (present in many plant species not botanically related) protease sensitive and less heat sensitive that mainly induce an oral allergy syndrome, while severe reactions are rare [49].

Therefore, the allergist approaching the MBD should know the chemical, physical and immunological characteristics of all allergenic families, their biodegradability, cooking/heat resistance/sensibility etc. The stability/lability of a molecule (along with the clinical history) helps the clinician to evaluate the risk of systemic versus local reactions. Stable allergens are generally associated with severe systemic reactions, whereas labile allergens are associated with low/mild reactions and cooked food is often tolerated.

Moreover, it is essential to know to which family the various molecules belong and their structural similarity within the family (generally characterized by a greater than 50–70 % sequence homology).

In the above-mentioned example of apple, MBD can distinguish between fruit allergy due to LTP sensitization and a pollen-related apple allergy. Sensitization to Mal d 3 (an LTP protein) indicates a fruit allergy where peach is often the primary sensitizer [50, 51]. Sensitization to Mal d 1 (a PR-10 protein) is seen in birch-pollen allergic patients and is caused by cross-reactivity with the main birch allergen Bet v 1 [52, 53]. The presence of IgE antibodies to profilin (e.g. Mal d 4, homologous of Phl p 12) is indicative of an apple allergy related to a grass-pollen sensitization [53, 54]. Patients with IgE antibodies to Mal d 2 and 3 (LTP stable proteins) are at higher risk of developing systemic reactions. IgE antibodies to Mal d 1 and/or profilin and not to Mal d 2 and 3 suggest that predominantly local oral symptoms may occur. Apple-allergic patients sensitized to Mal d 3 may tolerate peeled apples. Apple-allergic patients sensitized to Mal d 1 and/or profilin (that are labile proteins) may often tolerate cooked apples.

MBD is a complex area, but as it provides new and relevant information for the allergist, it will soon become a standard tool for the diagnosis of FA. Educational programs for allergists on the use and interpretation of MBD are needed [55].

In vitro MBD is defined as single or multi-plexed IgE assay microarray. By the single-plexed diagnostics the choice of the components to be tested is relies on the allergist’s judgment, based on the patient’s sIgE profile. In poly-sensitized patients, a complete recognition of the IgE profile might require a large number of assays. In these cases, it may be reasonable to use the multi-plexed allergen microarray (AMA) that allows for the detection of specific reactivity to over 100 allergen components. The most popular form (the Immuno-Sorbent Allergen Chip—ISAC) currently contains inhalants, foods, latex and insect venom. Despite AMA not being a quantitative assay, the correlation between the results of microarrays and the results of sIgE tests are largely super-imposable. Thus AMA is suitable in both paediatric and adult serum samples when the number of molecular components to be tested using single-plexed methods is too high to be cost-convenient or when the need for extensive research of sensitization is required [20]. This is particularly true in highly complex patients presenting symptoms of a cross-sensitization to inhalant and food and clinical evidence of food allergy. AMA is a powerful in vitro test that requires specific expertise but provides a very large amount of information to the allergist.

MBD diagnostics, especially microarray, are expensive compared with traditional tests, unless a single test is considered. Economic considerations may influence the decision of using a single or multiplex approach in individual patients. Using the microarray diagnostics allows for the performance of a broad-spectrum analysis of a patient’s IgE profile with a small blood sample. It may reveal unanticipated sensitivities, possibly to potentially harmful molecules, making the interpretation of such sensitization difficult in the case of a clinically silent history, but giving the allergist the chance to investigate other hypersensitivities and to alert the patient towards possible risks. This clearly demonstrates that in vitro diagnostics, including MB, should be evaluated within the framework of a patient’s clinical history, since allergen sensitization does not necessarily imply clinical responsiveness.

### Third-level methods

#### Oral provocation test (OPT)

OPTs are the most reliable tests in the diagnosis of clinically relevant IgE associated food allergies once allergen specific IgE has been detected. The OPT remains the “gold standard” to establish or exclude the liability of a particular food in causing an adverse reaction [56–61]. The actual value of this method is its functional result. Indeed, only foods causing a clinical evidence of allergy are considered positive. When first and second-level methods have been unable to indicate the food that is responsible for the symptoms, the clinical relevance of a detected sensitization may also be investigated by a targeted elimination diet to perform before the OPT [28]. Furthermore, if multiple triggers are suspected, the elimination diet can help in selecting food to be tested through the OPT, which remains the most important diagnostic tool in food allergy diagnosis. Once the diagnostic work-up has been concluded, the elimination diet of the culprit food/s usually represents the treatment for known food allergies, as well as educating the patient about proper food preparations and the risks of occult exposure [28, 29]. Ongoing investigations are currently evaluating the role of food immunotherapy as a potential FA therapy, to be performed by highly skilled specialists in appropriate settings [29, 62].

The OPT is a third-level procedure that should be carried out when previous diagnostic levels were unable to give sufficient information for the clinical diagnosis [60]. During the patient follow-up, OPT is useful in detecting an acquired tolerance for the specific food. The functional identification of the causative food allows one to avoid its assumption as well as the establishment of unnecessary rigid diets. Due to the potential risk of severe adverse events, the test has to be performed in a hospital setting with personnel trained in resuscitation procedures and the availability of emergency drugs.

The indications of OPT [61, 63–65] are: (a) to identify the food responsible for acute reactions, or to monitor the unexpected tolerance in case of a history of allergy; (b) to determine the offending food in chronic conditions such as atopic dermatitis or eosinophilic esophagitis; (c) to expand the diet in subjects with multiple dietary restrictions; (d) to establish the degree of tolerance to cross-reactive foods and to establish possible acquisition of a spontaneous tolerance to food.

The contraindications are: (a) previous severe anaphylactic reactions (especially recent); (b) level of specific IgE exceeding the cut-off for which there is a high probability that the oral test is positive; (c) reactivity to individual molecules identified with the MBD that indicate a possible severe reaction; (d) reactions occurred during the performance of the SPT and (e) a progressive systemic disease, in particular when the patient is taking medications that could mitigate (antihistamines, corticosteroids) or amplify (β-blockers, ACE inhibitors, NSAIDs, etc.) the reaction [63, 64].

The test consists of gradually increasing doses of the appropriately diluted food, starting from the lowest dose and checking the presence of relevant symptoms. The test can be performed in three different settings [63]:

*Open OPT* is used for immediate reactions when the risk of severe reaction is reduced. It can be performed on an outpatient basis with a simplified protocol of administration and an observation time of about 2 h. It can be strongly influenced by the age and by the subject’s psychological behaviour. If negative, the food can be reintroduced into the diet. In the case of suspected positive reaction, it should be checked in a double-blind OPT setting.

*Single-blind placebo OPT (SBP-OPT)* it consists of two sessions, one with placebo and one with the suspected food. When a strong psychological component is suspected, the placebo should be tested first. The patient undergoing the SPB-OPT is informed that the food may or may not be present in the administered dose. If the answer is negative or positive symptoms are observed, it is not necessary to continue the investigation. Repeated sessions with placebo or suspected food are useful for the confirmation of vague symptoms. In the case of positivity with placebo, a DBP-OPT will be necessary. In the case of a negative result, the tolerated food must be ingested in its natural form 2 h after or on the day after the test. The tolerance should be checked with follow-up.

*Double–blind placebo controlled test (DBPCT)* the gold standard. The foods to be tested are prepared by professional personnel not involved in the clinical examination. Placebo and food must have a very similar look and taste. Only when the test is completed can the doctor and patient know the pattern of administration and discuss the results [66].

### Fourth-level methods

#### Basophil activation test (BAT)

The BAT can be used in the study of IgE (and non-IgE) mediated allergic reactions [67, 68]. The rationale of this test is the change in the phenotype of activated basophils after in vitro incubation of the patient’s whole blood with the allergen. The BAT is a useful complementary tool to the in vitro diagnosis of FA caused by milk, egg, peanut and wheat [69, 70] when IgE may be involved. Interestingly, a recent study of 20 peanut-allergic children showed that when basophils were stimulated with decreasing doses of allergens until threshold sensitivity was reached, 19 were negative to peanut but 17 were positive to rAra h 8, suggesting that the children sensitized to Ara h 8 but not peanut storage proteins may be at risk of systemic allergic reactions, especially when eating large amounts of peanuts [70].

**Fig. 1:**
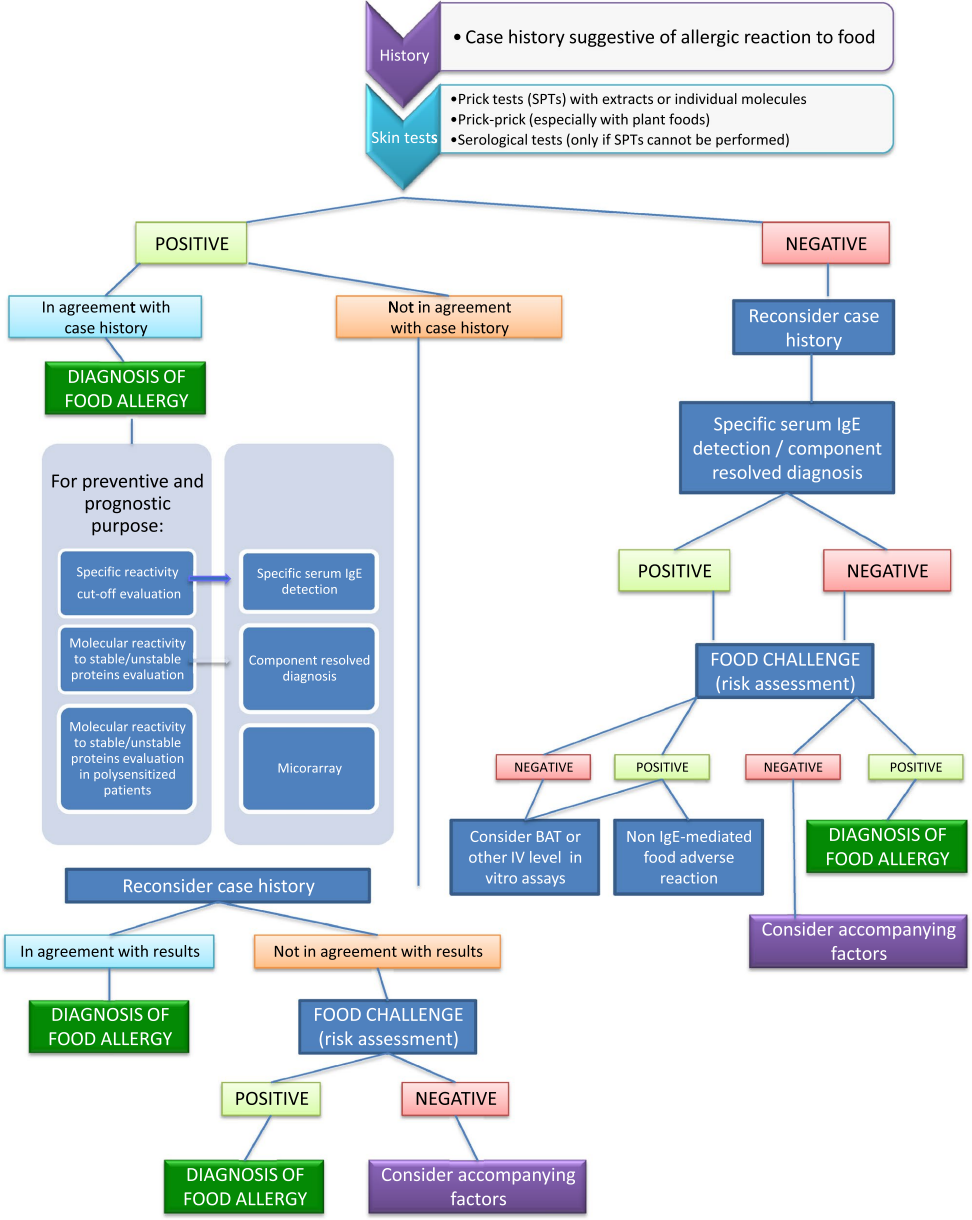
Flow chart for the diagnosis of food allergy

Reactions unrelated to IgE may also be assessed by BAT, as evidenced for wine and beef [71, 72]. Recently it was also used in the decision-making process for the reintroduction of milk in children allergic to casein [73]. Today, BAT is the only assay that mimics, in the test tube, what happens in vivo. After an extensive validation, BAT should distinguish sensitization from a clinical allergy. The method still suffers some critical issues that can make it a routine test only in specialized laboratories (Fig. 1).

### Complementary alternative tests

Frequently, patients undergo complementary/alternative tests after a negative response to a common diagnostic work-up or when non specific symptoms predominate (e.g. migraine, abdominal discomfort, chronic urticaria or other skin abnormalities, chronic fatigue, weight gain or lack of success in weight loss diets), which are erroneously classified as “food allergy” [74]. It represents a common diagnostic label suggested by physicians without specific expertise in the field of FA mechanisms and food-related disorders [75, 76].

The most common (not validated) alternative diagnostic techniques are:

In vivo:

- Electrodermal tests: they measure the change in the skin’s electrical conductance once the subject has been exposed to an allergenic substance through specific devices [75].
- Kinesiology: it registers the decreased strength of muscular contraction related to contact with an allergen [75].
- Provocation/neutralization testing: it identifies the onset of “untoward effects” provoked by the administration (intradermal or sublingual) of allergenic substances [76]. The same technique is used as a therapeutic tool.

In vitro:

- Leukocytotoxic tests: they detect the shape/volume abnormalities of peripheral leukocytes when an allergen in a solid and not measurable phase comes into contact with them [77].

A direct comparison between such tests and gold standard methods has so far failed in all cases to demonstrate their validity [78]. Their use is strongly discouraged.

## Conclusion

The diagnosis of FA is an integrated procedure that can be carried out in different steps (Fig. 1). Some patients can be diagnosed easily by the use of first- and second-level tests, while complex patients, with poly-sensitization and pollen-food syndromes, frequently require a third-level approach. In recent years, the diagnostic assays for FA have been significantly expanded and standardized tools and procedures are now available to the allergist.

Currently, demanding issues are related to FA diagnosis: (1) Identified food(s) should be excluded from the diet; (2) the patient must be properly informed about the relative risk of ingesting the sensitizing foods, even inadvertently as hidden foods in different preparations; (3) the allergist should explain all preventive and curative measures to be taken in case of allergic reactions, including potential medical urgency. In particular, the patient must be informed of the possibility that certain concurrent conditions could favour the onset of FA. This involves a great deal of renewed research specialists and experts on the subject to be able to respond appropriately to the diagnostic and therapeutic needs of patients.

## Abbreviations

AMA: allergen microarray
APT: atopy patch test
BAT: basophil activation test
DBPCT: double-blind placebo-controlled test
FA: food allergy
FDEIA: food dependent exercise induced anaphylaxis
ISAC: immuno-sorbent allergen chip
LTP: lipid transfer protein
MBD: molecular-based diagnosis,
NSAIDs: non-steroidal anti-inflammatory drugs
OPT: oral provocation test
PR-10: pathogenesis-related-10
P + P: prick + prick
SBT-OPT: single-blind placebo oral provocation test
sIgE: specific IgE
SPT: skin prick test
tIgE: total IgE
