# Erratum to: Guidelines for the use and interpretation of diagnostic methods in adult food allergy

**DOI:** 10.1186/s12948-015-0037-5

**Published:** 2015-12-21

**Authors:** Donatella Macchia, Giovanni Melioli, Valerio Pravettoni, Eleonora Nucera, Marta Piantanida, Marco Caminati, Corrado Campochiaro, Mona-Rita Yacoub, Domenico Schiavino, Roberto Paganelli, Mario Di Gioacchino, On behalf of the Food Allergy Study Group (ATI) of the Italian Society of Allergy, Asthma and Clinical Immunology (SIAAIC)

**Affiliations:** SS Allergology and Clinical Immunology, S. Giovanni di Dio Hospital, Florence, Italy; Respiratory Diseases and Allergy, University of Genoa, IRCCS AOU S. Martino-IST, Genoa, Italy; Allergology and Immunology Unit, IRCCS Ca’ Granda, Osp. Maggiore Policlinico, Milan, Italy; Servizio di Allergologia, Policlinico Gemelli, Rome, Italy; Allergy Unit, Verona University and General Hospital, Verona, Italy; Department of Allergy and Clinical Immunology, IRCCS San Raffaele Hospital, Milan, Italy; Department of Medicine and Science of Ageing, G. d’Annunzio University, Chieti, Italy; Unit of Allergy and Immunotoxicology, CeSi, “G. d’Annunzio” University Foundation, Chieti, Italy

## Erratum to: Clin Mol Allergy (2015) 13:27 DOI 10.1186/s12948-015-0033-9

After the publication of this work [1] some errors were noticed in Figure 1. The corrected figure is provided below. The original conclusions will not be affected by this Erratum.

**Fig. 1:**
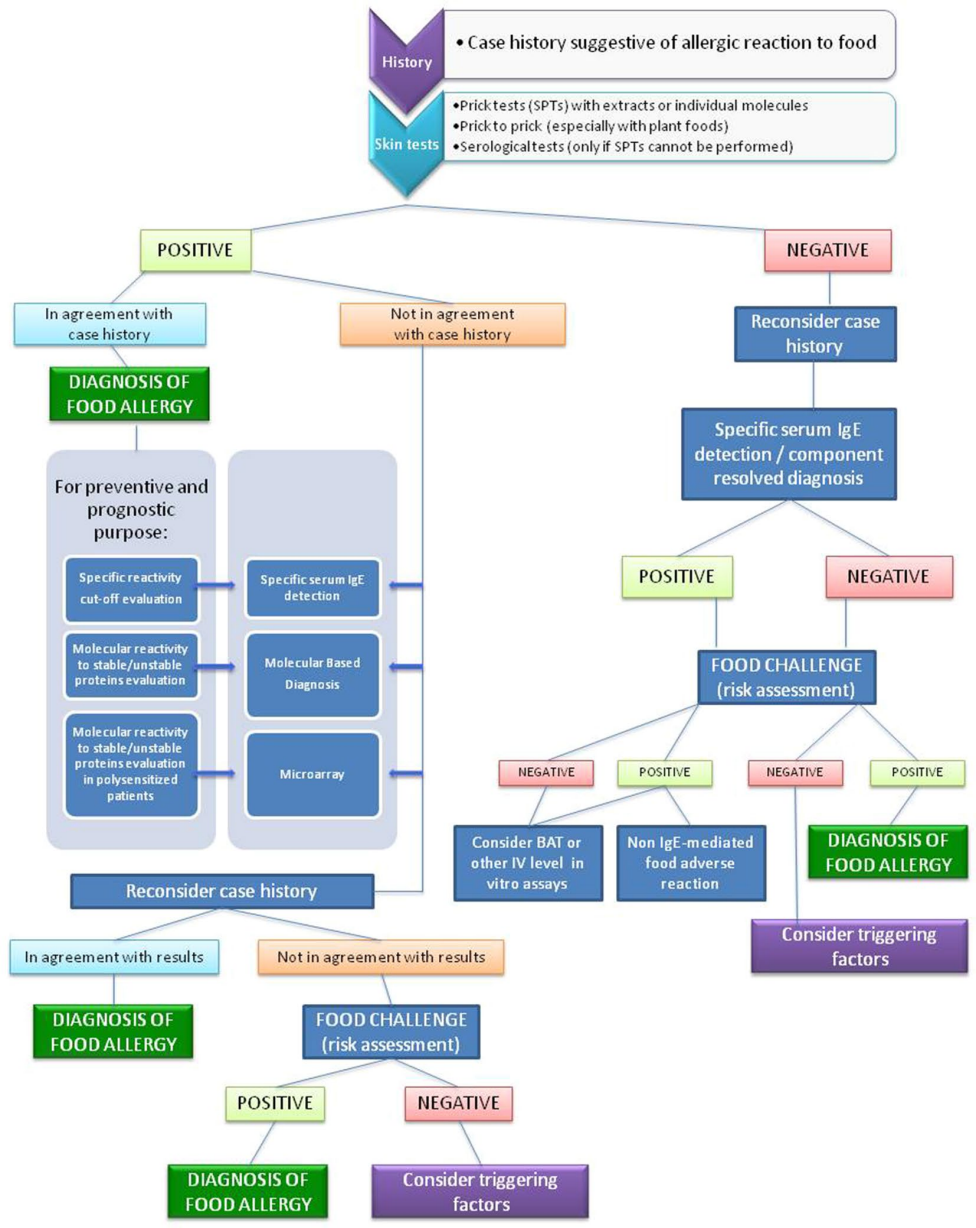
Flow chart for the diagnosis of food allergy
